# Orthogonal Chirp Coded Excitation in a Capacitive Micro-machined Ultrasonic Transducer Array for Ultrasound Imaging: A Feasibility Study

**DOI:** 10.3390/s19040883

**Published:** 2019-02-20

**Authors:** Bae-Hyung Kim, Seungheun Lee, Kang-Sik Kim

**Affiliations:** 1 Samsung Advanced Institute of Technology, Samsung Electronics Co. Ltd, Suwon-si, 16678, Korea; 2 Department of Mechanical, Automotive and Robotics Engineering, Dong-eui University, Busan 47340, Korea; sh.lee@deu.ac.kr; 3 Health & Medical Equipment Department, Samsung Electronics Co. Ltd, Suwon-si, 16678, Korea; kskim7@gmail.com

**Keywords:** CMUT, coded excitation, orthogonal chirps, simultaneous transmission multiple-zone focusing, frequency division multiple access

## Abstract

It has been reported that the frequency bandwidth of capacitive micro-machined ultrasonic transducers (CMUTs) is relatively broader than that of other ceramic-based conventional ultrasonic transducers. In this paper, a feasibility study for orthogonal chirp coded excitation to efficiently make use of the wide bandwidth characteristic of CMUT array is presented. The experimental result shows that the two orthogonal chirps mixed and simultaneously fired in CMUT array can be perfectly separated in decoding process of the received echo signal without sacrificing the frequency bandwidth each chirp. The experimental study also shows that frequency band-divided orthogonal chirps are successfully compressed to two short pulses having the −6 dB axial beam-width of 0.26- and 0.31-micro second for high frequency and low frequency chirp, respectively. B-mode image simulations are performed using Field II to estimate the improvement of image quality assuming that the orthogonal chirps designed for the experiments are used for simultaneous transmission multiple-zone focusing (STMF) technique. The simulation results show that the STMF technique used in CMUT array can improve the lateral resolution up to 77.1% and the contrast resolution up to 74.7%, respectively. It is shown that the penetration depth also increases by more than 3 cm.

## 1. Introduction

Coded excitation, which has employed pulse compression techniques used in radar and sonar systems for decades, can improve the signal to noise ratio (SNR) of medical ultrasound imaging by increasing average power without affecting instantaneous peak power [1,2,3,4]. Although there are many known compression waveforms for coded excitation such as maximal length sequences, Barker codes and linear frequency modulation (FM) signals called chirps, it has been known so far that the coded excitation using Golay and chirp coded signals have a good performance with respect to the image quality and the system requirement for medical ultrasound imaging [5,6,7,8,9]. Modulated excitation signals using Golay sequences and/or chirps having mutually orthogonal property have been proposed to increase not only the SNR and/or the penetration depth but also the frame rate and spatial resolution [8,9,10,11]. Recently, multi-line and multi-plane wave transmission beamforming using the orthogonal coded excitation have been proposed for fast ultrasound imaging [12,13,14,15].

As an approach to achieve high lateral resolution, synthetic transmit aperture techniques that can provide two-way dynamic focused beam fields in both transmission (Tx) and reception (Rx) have been studied and developed [16,17,18]. However, since several transmission and reception processes are required to make a scan-line of an image which results in long data acquisition time, the synthetic transmit aperture technique has a problem of phase distortion caused by motion artefacts [18,19].

To improve the lateral resolution of traditional ultrasound B-mode image without reducing the frame rate, simultaneous transmission multiple-zone focusing (STMF) techniques using orthogonal chirp or Golay coded excitation were introduced [20,21,22,23,24,25,26]. In the STMF method using orthogonal chirps, orthogonal chirp coded signals are simultaneously fired on multiple depths, where each chirp is focused at a different depth in one transmission event [22,23,25,26]. Then, ultrasound images are reconstructed through a decoding process of orthogonal sub-band divided chirps designed within the frequency bandwidth of a transducer. However, the frequency bandwidth of transducer limits the frequency bandwidth of sub-band chirps which results in a degraded axial resolution at each depth in the STMF technique. If the frequency bandwidth of the transducer is large enough so that each sub-band signal bandwidth is comparable to the conventional short pulse, the STMF method is feasible to achieve the dynamic Tx focused imaging without reducing the axial resolution.

Meanwhile, it has been known that capacitive micro-machined ultrasonic transducer (CMUT) technologies provide unique opportunities to minimize the size and cost of ultrasound scanners by fabricating and interconnecting the front-end circuits into CMUT array with higher performance in terms of features and image qualities [27,28,29]. Moreover, CMUT has been pointed out as a promising technology for large range of applications including medical diagnosis or treatment [27,30]. This CMUT array provides relatively broader frequency bandwidth than that of other ceramic-based ultrasonic transducer arrays [31]. With the goal of developing a differentiation technique to utilize the wide bandwidth characteristic of the CMUT array, a frequency division multiple transmission method with CMUTs was previously introduced [32]. The approach was a FDMA (frequency division multiple access) based STMF technique using orthogonal chirp coded excitation to efficiently utilize the characteristic of the CMUT’s broader frequency bandwidth.

Hence, the aim of this study is to demonstrate the feasibility that the 2-D CMUT-on-ASIC array can maximize the advantage of FDMA based STMF technique using orthogonal chirps in both simulation and experimental setups. In this paper, an experimental study on orthogonal chirp coded excitation in CMUT array is conducted which is a world-first work. In the experimental study, 2-D CMUT array integrated with front-end ASICs called 2-D CMUT-on-ASIC array which was previously developed and introduced is used [33,34,35,36,37,38]. A simulation study using Field II [39] and a tissue mimicking phantom is also presented to estimate the improvement of image quality and to provide the optimal solution among various combinations of Tx foci for the STMF technique using orthogonal chirps designed within the frequency bandwidth of CMUT array. Due to the complexity of transmitter for chirp coded excitation that arbitrary waveform generator and power amplifier are required, the phantom and in vivo study for this technique using a complete 2-D CMUT-on-ASIC arrays integrated with the ultrasound system is not available as of now. The objective of simulation study is to show preliminary study results for estimating B-mode image quality before the STMF technique using orthogonal chirp coded excitation in CMUT array is implemented into an ultrasound system. However, for more practical study, the actual impulse response of CMUT array is measured and reflected on the simulation and experimental studies. In-depth and extensive discussions about the experimental and simulation results are presented.

## 2. Orthogonal Chirp Coded Excitation

### 2.1. STMF Method Using Orthogonal Chirps

**Figure 1:**
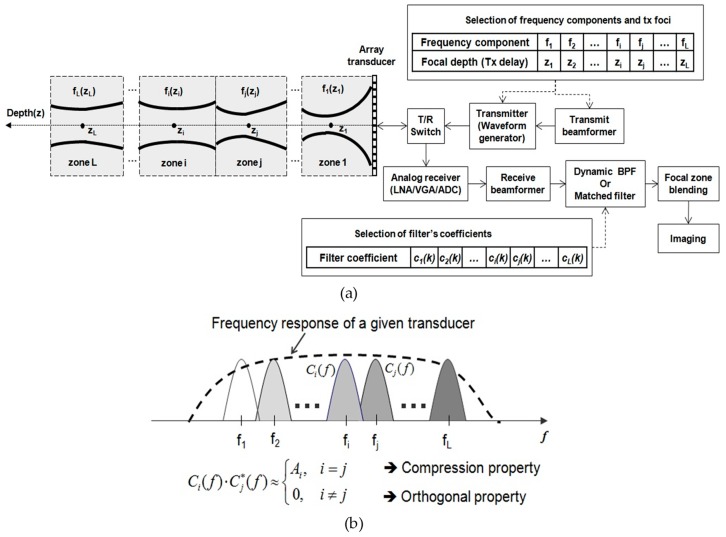
System block diagram of STMF method using frequency divided sub-band signals. (**a**) Illustration of the improvement of lateral beampattern; (**b**) design strategy of frequency band divided signals to have both compression and orthogonal property.

Figure 1 shows the system block diagram of the STMF technique using frequency division orthogonal signals to implement it into a medical ultrasound imaging system. The post-compression based coded excitation system and the zone-blending technique is adopted [24].

The improvement aspect of lateral beampattern is also illustrated in Figure 1a. As shown in Figure 1b, frequency divided *L* sub-band signals are designed inside the frequency bandwidth of a given transducer to have both compression and orthogonal properties. The mutually orthogonal signals are focused at *L* different depths and simultaneously transmitted with different transmission (Tx) delays. Tx beamformer calculates the Tx time delays for the *L* sub-band signals according to the Tx foci which are selected by the user. For example, the Tx focusing delays for each focal depth *i* and *j* are applied to the corresponding chirps $c_{i}$ and $c_{j}$, respectively. The aggregated *L* sub-band signals are transmitted to the medium. On reception (Rx), the received RF (radio frequency) echo signals are amplified in a low noise amplifier (LNA) and a variable gain amplifier (VGA) for the time-gain compensation (TGC), digitized by the analog-to-digital converters (ADCs), and dynamically focused in the Rx beamformer. The Rx beamformer output is then fed to correlators for matched filter processing. The *L* orthogonal sub-band signals which are individually focused in Tx are separated and compressed into each short pulse. The focused beams are combined to form an ultrasound image by the conventional zone-blending method. Therefore, the final B-mode image with improved image quality is provided by using a conventional echo-processing and digital scan conversion (DSC). An example of the composition result of multiple-zone focused beam-patterns is shown in the left side of Figure 1a. The solid line shows a blended lateral beam-pattern that *L* beam-patterns for the focal zones 1 to *L* are combined. In this way, the lateral resolution is improved by obtaining the multiple-zone focused Tx beam-pattern.

### 2.2. Encoding and Decoding of Orthogonal Chirps

This section describes the encoding and decoding process of the orthogonal chirp coded excitation for the FDMA based STMF technique. The linear FM signal whose instantaneous angular frequency varies linearly with time, called a chirp, can be defined as [40,41,42]:

(1) $$c\left(t\right)=A_{0}w\left(t\right)e^{j\left(w_{0}t+\frac{\mu t^{2}}{2}\right)}\;\left(j=\sqrt{-1},\;\mu =\frac{2\pi \Delta f}{T}\right),$$

where $w_{0}$ is the center angular frequency, μ is the rate of the instantaneous angular frequency change, Δf is the frequency bandwidth of the chirp, T is the time duration of the chirp, $A_{0}$ is the amplitude gain, and w(t) is a window function that determines the envelope of the chirp.

The matched filter output for the chirp is given by:

(2) $$c\left(t\right)⊗c^{*}\left(-t\right)=A_{0}^{2}e^{j\left(w_{0}t+\frac{\mu t^{2}}{2}\right)}\int_{-\infty }^{\infty }w\left(\tau +t\right)w^{*}\left(\tau \right)e^{jwt\tau }d\tau ,$$

where ⊗ and * denote the convolution and conjugate operation, respectively. Assuming frequency divided *L* sub-band chirps are given by $c_{i}\left(t\right)=w_{i}\left(t\right)e^{j\left(w_{i}t+\frac{\mu t^{2}}{2}\right)}\;\left(1\leq i\leq L\right)$ which are mutually orthogonal, the orthogonal and pulse compression property of the chirps can be expressed by:

(3) $$c_{i}\left(t\right)⊗c_{j}^{*}\left(-t\right)≅\{\begin{array}{cc} A_{i}\delta \left(t\right) & if\;1\leq i=j\leq L,\\ 0 & if\;otherwise, \end{array}$$

where $A_{i}$ is the gain in pulse compression which is proportional to the product of the time duration and frequency bandwidth of the chirp [22,25]. In the frequency domain, the orthogonal and pulse compression property of the frequency band-divided chirps is clearly defined as:

(4) $$C_{i}\left(f\right)\times C_{j}\left(f\right)≅\{\begin{array}{cc} A_{i} & if\;1\leq i=j\leq L,\\ 0 & if\;otherwise. \end{array}$$

In the STMF method, assuming the *L* orthogonal chirps are used for providing a Tx focused beam at *L* different depths in each scan direction, the aggregated orthogonal chirps using the Tx focusing delay $t_{i}$ of each chirp $c_{i}$ can be expressed as:

(5) $$g\left(t\right)=\overset{L}{\underset{i=1}{\sum }}c_{i}\left(t-t_{i}\right).$$

Then, the delayed RF-echo signal is given by:

(6) $$r\left(t\right)=g\left(t-\tau \right)=\overset{L}{\underset{i=1}{\sum }}c_{i}\left(t-t_{i}-\tau \right),$$

where τ is the delay of the received RF echo signal and the amplitude scaling related to ultrasound wave propagation is not included. The received RF echo signal is separated into pulse-compressed waveforms of each chirp by correlating with *L* respective orthogonal chirps using the orthogonal property of Equation (3), yielding:

(7) $$r_{i}\left(t\right)⊗c_{i}^{*}\left(-t\right)≅A_{i}\delta \left(t\right),\;1\leq i\leq L.$$

Finally, the *L* compressed chirps are independently focused and then each beam having different focal depth is combined to provide the dynamically transmit-focused beam along each scan line. The STMF method can easily be extended for an arbitrary number *L*, and the *L* orthogonal chirps are designed based on frequency division within a given transducer bandwidth.

## 3. Methods

### 3.1. Experimental Setup for Orthogonal Chirp Coded Excitation in CMUT Array

**Figure 2:**
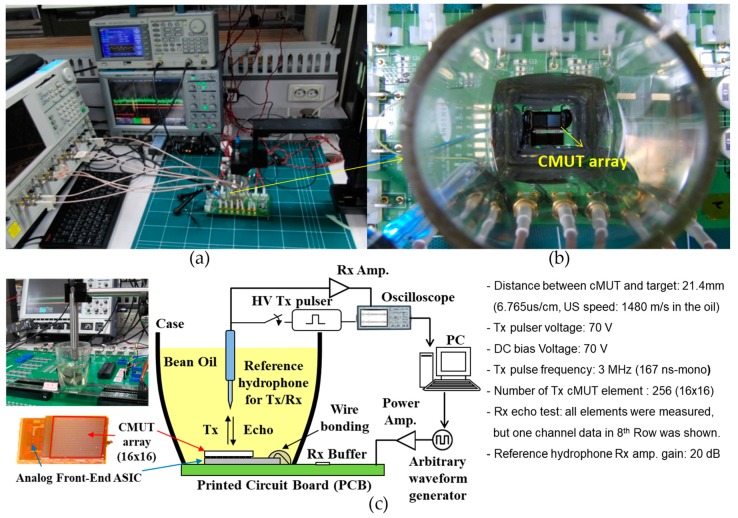
Experimental setup for CMUT array. Photos of (**a**) experimental environment and (**b**) 2-D CMUT-on-ASIC arrays, and (**c**) detailed block diagram and experimental conditions for Tx/Rx test with reference hydrophone.

An experimental setup is shown in Figure 2. Figure 2a,b display the photos of experimental environment and the 2-D CMUT-on-ASIC array, respectively. Figure 2c shows the detailed block diagram and key experimental conditions for the measurement in Tx and Rx test. Orthogonal chirps were designed and generated using MATLAB (The Mathworks Inc., Natrick, MA, USA) in PC and down-loaded to arbitrary waveform generator (AWG). The chirps were amplified by a linear power amplifier, and then transmitted to the 2-D CMUT-on-ASIC array in soy bean oil. After the chirps were fired from the 2-D CMUT-on-ASIC array and propagated through the bean oil, the reflected signal from a hydrophone placed at 21.4 mm distance was captured by the 2-D CMUT-on-ASIC array and the one-way travelled signal was also received and captured by the hydrophone to double-check the response. Then, the received signal was measured, and finally saved into the oscilloscope as a digitized data. The pulse-compression and separation with the correlators were performed in PC.

For the experiment, all channels for 256 elements were used for plane wave Tx and were measured for Rx echo test. As the property of the CMUT array and ASIC device was introduced in [33,34] and the CMUT-ASIC array was utilized in previous works [35,36,37,38], all the elements’ properties and those measurement results are not described here and readers should consult the references for details.

### 3.2. Simulation Setup

In the simulation study, the STMF method using the orthogonal chirps designed within the frequency bandwidth of CMUT array is compared to the conventional single-zone focusing (SF) method using a pulsed-wave. The mimicking tissue phantom used in B-mode simulation contains nine point targets, five anechoic cysts located at −10 mm in lateral direction with a start depth of 30 mm, and five hyperechoic masses located at 10 mm in lateral direction with a start depth of 30 mm, and 200,000 random scatters for speckles. The anechoic and hyperechoic targets are separated by 10 mm in depth. All anechoic cysts have a gain of 0, and an increased radius by 0.5 mm from 1 mm to 3 mm in depth. All hyperechoic masses have an amplitude gain of 20 dB compared to the surrounding speckles, and a decreased radius by 0.5 mm from 3 mm to 1 mm in depth.

## 4. Results and Discussion

### 4.1. Measurement Result of CMUT Array

The design details about the 2-D CMUT-on-ASIC array and the property of all the elements are not described here for succinctness and one can refer to [33,34,35,36,37,38] for details. Instead a RF echo data of one element measured in 8th row and column, which is in the vicinity of the center position of the CMUT array (16 × 16 elements) is shown to demonstrate the impulse response and Tx/Rx pulse echo test of the 2-D CMUT-on-ASIC array.

#### 4.1.1. Impulse Response

**Figure 3:**
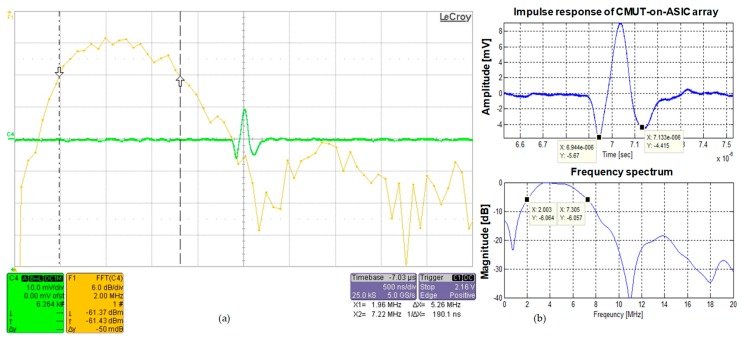
The impulse response (green line in (**a**) and upper figure in (**b**)) and the frequency spectrum (yellow line in (**a**) and lower figure in (**b**)) of the CMUT-on-ASIC array: (**a**) a picture captured from oscilloscope; (**b**) off-line plot using MATLAB with the impulse response data.

Figure 3a,b show the impulse response (green line in Figure 3a and upper plot in Figure 3b) and frequency spectrum (yellow line in Figure 3a and lower plot in Figure 3b) of the 2-D CMUT-on-ASIC array. Figure 3a is a picture captured from the oscilloscope (Teledyne LeCroy, Chestnut Ridge, NY, USA) and Figure 3b is off-line time (top) and frequency (bottom) plots of captured impulse response data using MATLAB. The measured center frequency and −6 dB frequency bandwidth of the 2-D CMUT-on-ASIC array were 4.66 MHz and 5.31 MHz, respectively. The fractional bandwidth was 114% of the center frequency, which is about twice the frequency bandwidth of other ceramic-based conventional ultrasonic transducers. Two orthogonal sub-band chirps within −6 dB frequency bandwidth ranged from 2 MHz to 7.31 MHz were designed to satisfy the orthogonal property and were used for simultaneous transmission in CMUT array at two different focus depths. The orthogonal chirps were generated in PC using MATLAB to demonstrate that the received signals are successfully separated into two compressed signals. The two orthogonal sub-band chirps were utilized for the simulation and experiment of the next sections to verify the feasibility of the FDMA based STMF technique using the orthogonal chirp-coded excitation in CMUT array.

#### 4.1.2. Tx and Rx Pulse-Echo Test

**Figure 4:**
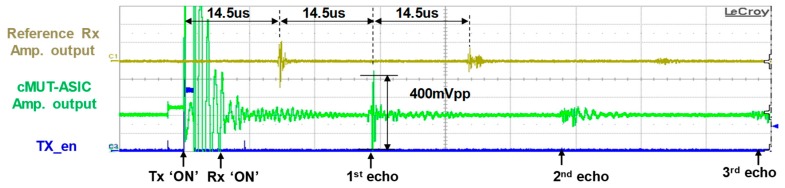
Measurement result for Tx and Rx pulse-echo test in CMUT array.

Figure 4 shows Tx and Rx pulse-echo test result. As the distance between CMUT and the target (reference hydrophone) is 21.4 mm and the speed of ultrasound in oil is 1480 m/sec, one-way trip time is about 14.46 micro second. We can see this in gold line of Figure 4 that shows the output signal of Rx amplifier of reference hydrophone. The round-trip echo reflected from reference hydrophone is shown in green line of Figure 4 which is the Rx amplifier output signal of CMUT-ASIC. We can also see the second signal reflected from CMUT in gold like of Figure 4 which is measured in reference hydrophone. The second and third pulse-echo signals are also observed in green line of Figure 4.

### 4.2. Design of Two Orthogonal Chirps in CMUT Array

**Figure 5:**
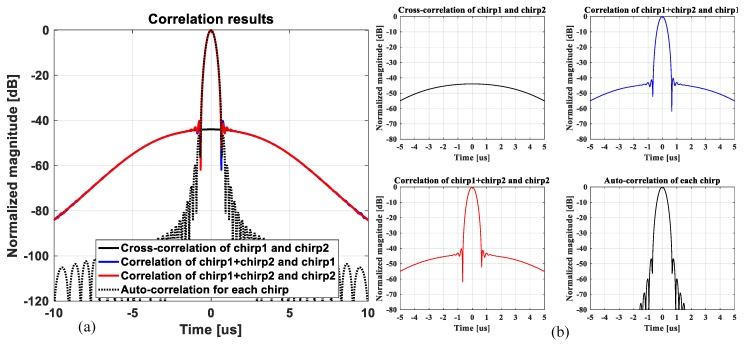
(**a**) Correlation results in time to show the cross-correlation of the two chirps (black solid line), the correlation of combined two chirps and chirp1 (blue solid line), the correlation of combined two chirps and chirp2 (red solid line) and the auto-correlation of each chirp (black dotted line) and (**b**) those zoomed-in and separate plots.

The −6 dB fractional bandwidth of the CMUT-on-ASIC array is 114% of the center frequency of 4.66 MHz as shown in Figure 3. Thus, for the B-mode image simulation, two sub-band chirps with the −6 dB frequency bandwidth of ranged from 1.95 MHz to 4.41 MHz ($c_{1}\left(t\right)$, lower frequency band-increasing linear FM signal) and from 7.36 MHz to 4.89 MHz ($c_{2}\left(t\right)$, higher frequency band-decreasing linear FM signal) are designed and utilized to reflect the measured impulse response of the CMUT-on-ASIC array. In medical ultrasound imaging, it is necessary that the maximum main-lobe to side-lobe level ratio of an auto-correlation result of each chirp should be below −40 dB as the dynamic range of tissue that differentiates from back scattering noise is known as about 40 dB [25,26]. To meet the orthogonal property between the two chirps, this requirement needs to be applied to a cross-correlation result. The two chirps are designed to have the maximum value of cross-correlation between the two chirps be lower than −44 dB relative to the maximum value of auto-correlation of each chirp as shown in Figure 5. 

Figure 5 displays the correlation results in time of the two chirps designed for the simulation study. The cross-correlation of the two chirps, the correlation of combined two chirps ($c_{1}\left(t\right)+c_{2}\left(t\right)$) and chirp1 ($c_{1}\left(t\right))$, the correlation of combined two chirps ($c_{1}\left(t\right)+c_{2}\left(t\right)$) and chirp2 ($c_{2}\left(t\right))$ and the auto-correlation of each chirp are shown all together in Figure 5a. Those zoomed-in plots are separately displayed in Figure 5b.

Tx foci of the two chirps are set to six (6) combinations as seen in Table 1. In the conventional SF method, a pulsed wave of the center frequency of 4.66 MHz and −6 dB frequency bandwidth of 2.52 MHz was used. The Tx focus in SF method was fixed at 40 mm. The transducer model used was a 192-element and 250 micron-pitch linear array transducer with the center frequency of 4.66 MHz and the −6 dB fractional bandwidth of 5.3 MHz. For a linear array imaging, a 64 active Tx/Rx channel system was used. A Hanning window was used in the excitation signal for both the STMF method and the SF method. The main reason why Hanning window was used in this work is that it is widely used for the chirp window as shown in the references [10,11,25,26] and it meets the criteria for the suppressed cross-correlation of the two chirps in term of the orthogonal property. Furthermore, whatever window function is used, the implementation of multi-phase signal for chirp-coded excitation is a major issue in ultrasound system as the apodization makes the needs for a multi-level transmitter which generally requires a power amplifier and arbitrary waveform generator and a multi-level decoder for matched filtering. Although the use of different window function can make a different correlation result, it is not a scope of this work. The study on the use of various window functions for orthogonal chirps could be a scope of further study. One can also refer to the references for the recent studies about the window function [43,44,45]. The radio frequency (RF) data was sampled temporally at 80 MHz. The frequency-depth dependent attenuation of 0.3 dB/MHz/cm in the medium was considered for the simulation. TGC was applied for compensating the attenuation in reconstructing of B-mode images. Simulation parameters are summarized in Table 1.

### 4.3. Experimental Results for Orthogonal Chirp Coded Excitation in CMUT Array

#### 4.3.1. Pulse Compression of Two Orthogonal Chirps in CMUT Array

**Figure 6:**
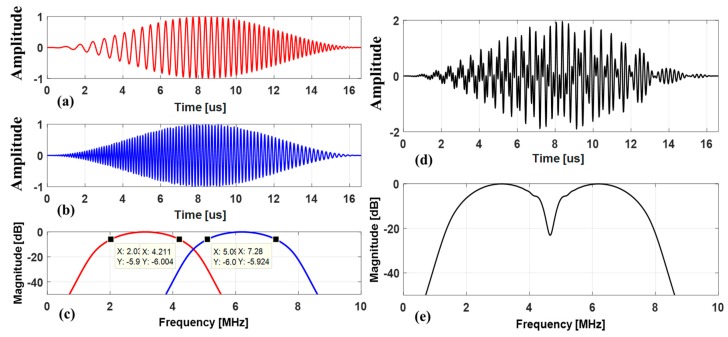
Time waveforms and frequency spectrums of frequency band divided two sub-band chirps with mutually orthogonal property. Time waveforms of (**a**) lower frequency band chirp-increasing linear FM over time (chirp1: $c_{1}\left(t\right)$) and (**b**) higher frequency band chirp-decreasing linear FM over time (chirp2: $c_{2}\left(t\right)$); (**c**) corresponding frequency spectrums of lower frequency chirp and higher frequency chirp; (**d**) transmission waveform of added two sub-band chirps; (**e**) the corresponding frequency spectrum of the added two chirps.

Two sub-band chirps designed for the experiment are displayed in Figure 6. The lower frequency chirp, $c_{1}\left(t\right)$ was designed to linearly increase the instantaneous frequency with time and to have the −6 dB frequency bandwidth ranged from 2 MHz to 4.2 MHz. The higher frequency chirp, $c_{2}\left(t\right)$ was designed to linearly decrease the instantaneous frequency with time and to have the −6 dB frequency bandwidth ranged from 7.3 MHz to 5.1 MHz. The chirp waveforms and frequency spectrums are shown in Figure 6a–c. The aggregated chirps were used for simultaneously firing in CMUT array, whose waveform and frequency spectrum were shown in Figure 6d,e.

**Figure 7:**
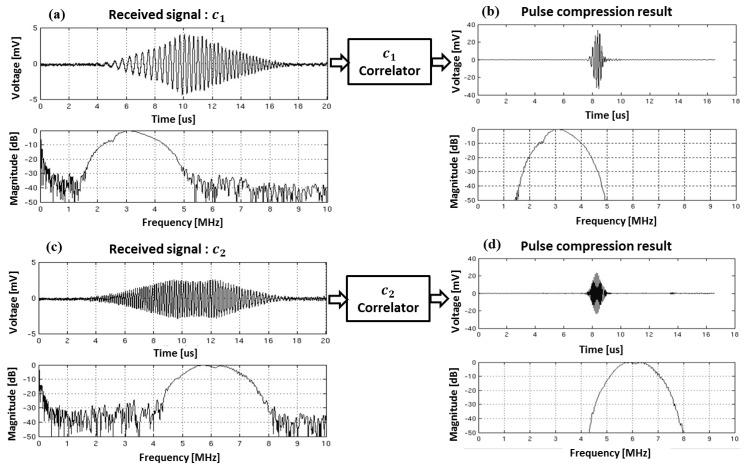
Time waveforms of received pulse-echo signal reflected from reference hydrophone and those frequency spectrums captured with the experimental setup shown in Figure 2 to verify pulse compression property of each sub-band chirps using correlators: (**a**) the received signal for firing lower frequency chirp $c_{1}\left(t\right);$ (**b**) pulse compression result using its correlator as the matched filter $c_{1}^{*}\left(-t\right)$; (**c**) the received signal for firing higher frequency chirp $c_{2}\left(t\right)$ and (**d**) pulse compression result using its correlator as the matched filter $c_{2}^{*}\left(-t\right)$.

Figure 7 displays the experimental results to illustrate the pulse-compressed waveforms of the received signals in a single chirp coded excitation. Figure 7a shows the received pulse-echo signal reflected from reference hydrophone and the corresponding frequency spectrum after transmitting $c_{1}\left(t\right)$. Figure 7b shows the pulse compression result obtained by using the matched filter coefficient of $c_{1}\left(t\right)$.

Figure 7c shows the received echo signal reflected from the hydrophone and the corresponding frequency spectrum after transmitting $c_{2}\left(t\right)$, and Figure 7d shows the pulse compression result obtained by using the matched filter coefficient of $c_{2}\left(t\right)$. As expected, through the decoding process using correlators, the time waveform of each chirp can be compressed into a short time signal with higher peak amplitude on the auto-correlation output. The gain in peak amplitude after matched filtering is about 8, which provides 9 dB gain in signal-to-noise-ratio (GSNR) [4,46]. Assuming the center frequency is 4.66 MHz and the average frequency-depth dependent attenuation coefficient in the mimicking tissue is 0.3 dB/MHz/cm, the round-trip attenuation of echo signal is 2.8 dB per cm [47,48]. Therefore, 9 dB gain in SNR increases the penetration more than 3 cm. It can be seen that the chirp coded excitation in CMUT array enhances the penetration by increasing the peak signal to noise ratio (PSNR).

The results also demonstrate that the coded excitation technique can be used in lower voltage transmission applications as well. The use of low voltage transmission in CMUT array can be an approach to solve an issue about the generation of harmonic components due to the intrinsic behavior of CMUT array [30].

Comparing the frequency spectrums between before and after matched filtering, it could be seen that the frequency bandwidth after matched filtering narrowed from Figure 7. The −20 dB frequency bandwidth after matched filtering of $c_{1}$ chirp was 2.4 MHz whereas it was 3.1 MHz before matched filtering. This shows that the −20 dB frequency bandwidth of $c_{1}$ chirp was reduced by 22.6%. The decrease in −20 dB frequency bandwidth of $c_{2}$ chirp was also 0.7 MHz from 3.2 MHz, which was 18.8% decline. The decrease in the frequency bandwidth after matched filtering can degrade the axial resolution by increasing the axial beam-width. This implies that the frequency bandwidth of compressed chirp becomes smaller than that of originally designed chirp, which should be reflected in consideration of the final image quality of axial resolution.

#### 4.3.2. Separation and Compression of Mixed Sub-band Chirps in CMUT Array

**Figure 8:**
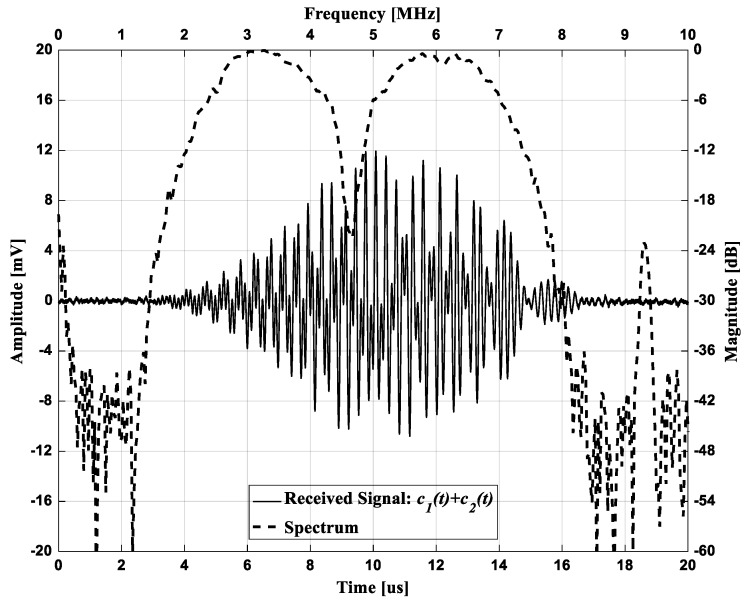
Time waveform of the received pulse-echo signal (solid line) after transmitting mixed two chirps $c_{1}\left(t\right)+c_{2}\left(t\right)$, and the corresponding frequency spectrum (dashed line).

In the experiment, two sub-band chirps considering different time delay were added and transmitted at the same time. Figure 8 shows the received pulse-echo signal reflected from the hydrophone after the mixed two chirps were transmitted and the corresponding frequency spectrum in dB scale. In the time waveform of Figure 8 (solid line), it seems impossible to find a form of each chirp because two chirps were added. However, when observing the frequency response of the received signal in Figure 8 (dashed line), it could be observed that the frequency component of each chirp was kept unimpaired. It could also be seen that a non-linear component in the spectrum around 9.31 MHz was generated at about 2 times of the center frequency of the transducer, 4.66 MHz. As the location of hydrophone was 21.4 mm, the distance between the CMUT and hydrophone was too short to generate a harmonic component due to non-linear propagation in the media. Also, as the center frequency of CMUT was 4.66 MHz, this non-linear component could be regarded as the generation of harmonic components due to the intrinsic behavior of CMUT array, rather than harmonic component of the transmitted waveform [30,49,50]. However, as the non-linear component was not included in the frequency bandwidth for STMF technique, the matched filter using correlator could remove this unwanted frequency component.

**Figure 9:**
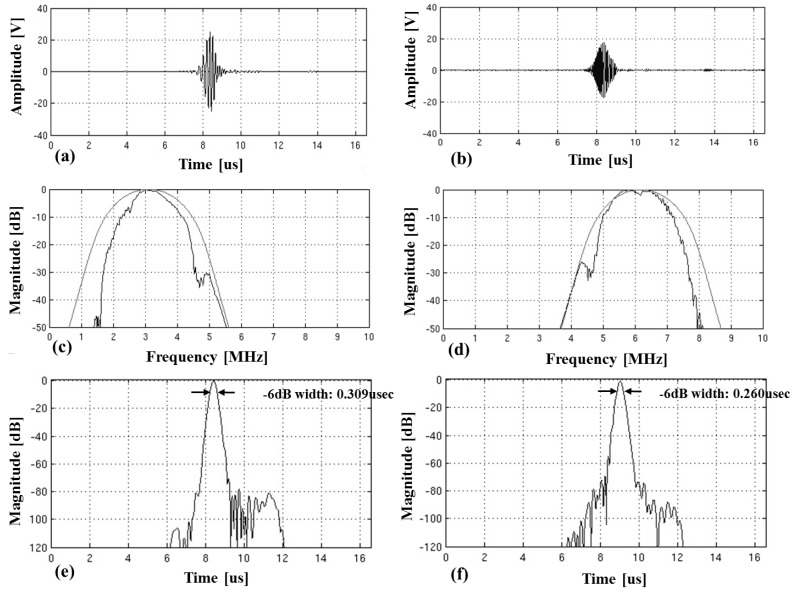
Separation and compression of two chirps received after simultaneously transmitting two orthogonal chirps. Time waveforms showing the results the received signal is correlated with (**a**) $c_{1}$ correlator and (**b**) $c_{2}$ correlator, corresponding frequency spectrums of (**c**) $c_{1}$ correlator (outer line) and correlation output (inner line) and (**d**)$\;c_{2}$ correlator (outer line) and correlation output (inner line), and corresponding envelop signals in dB scale of (**e**) $c_{1}$ correlation output and (**f**) $c_{2}$ correlation output.

Figure 9 shows the pulse compression and separation test results of the received pulse-echo signal after the mixed two chirps were transmitted. Figure 9a,b display the correlation outputs of the received signal with $c_{1}\left(t\right)$ and $c_{2}\left(t\right)$, respectively. Figure 9e,f show the corresponding envelopes in dB scale. Figure 9c,d show the frequency spectrum of the correlation outputs (inner line) and the frequency spectrum of the correlators (outer line) for $c_{1}\left(t\right)$ and $c_{2}\left(t\right),$ respectively. From Figure 9c, it can be seen that only the frequency component of $c_{1}\left(t\right)$ remained but that of $c_{2}\left(t\right)$ completely disappeared. In the same way, only the frequency component of $c_{2}\left(t\right)$ remained because $c_{1}\left(t\right)$ was completely removed as shown in Figure 9d. 

Figure 9e,f demonstrate that the envelopes of correlation outputs were compressed into two short pulses that provided the −6 dB axial beam-width of 0.31 and 0.26 micro-seconds, respectively. These pulse lengths are about 44% and 21% longer than 0.215 micro-second which is equivalent to cycles of Hanning windowed 4.66 MHz pulsed wave in the full width at half maximum (FWHM). From Figure 9, it was shown that the simultaneously transmitted orthogonal chirp-coded signals in CMUT array were successfully separated as each sub-band chirps, $c_{1}\left(t\right)$ and $c_{2}\left(t\right)$, and compressed into two short pulses.

### 4.4. B-Mode Image Simulation and Evaluation Results

**Figure 10:**
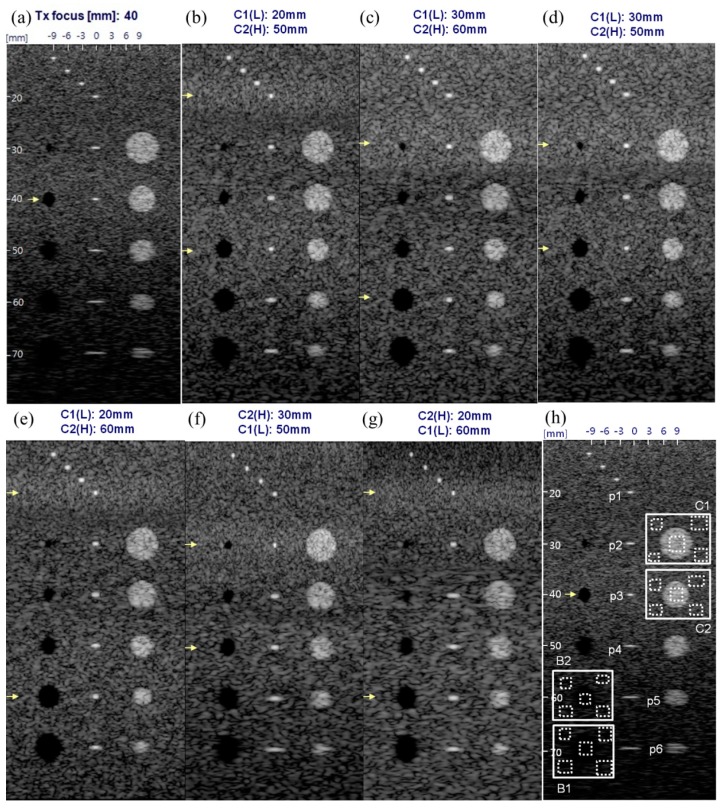
Computer generated B-mode images using a tissue mimicking phantom model (dynamic range: 60 dB). (**a**) The SF method: Tx focused at 40 mm, and STMF methods: Tx focused at (**b**) 20 mm for $c_{1}\left(t\right)$ (lower frequency chirp) and 50 mm for $c_{2}\left(t\right)$ (higher frequency chirp); (**c**) 30 mm for $c_{1}\left(t\right)$ and 60 mm for $c_{2}\left(t\right)$; (**d**) 30 mm for $c_{1}\left(t\right)$ and 50 mm for $c_{2}\left(t\right)$; (**e**) 20 mm for $c_{1}\left(t\right)$ and 60 mm for $c_{2}\left(t\right)$; (**f**) 30 mm for $c_{2}\left(t\right)$ and 50 mm for $c_{1}\left(t\right)$; and (**g**) 20 mm for $c_{2}\left(t\right)$ and 60 mm for $c_{1}\left(t\right)$; (**h**) marks of point targets (P1~P6), black holes (B1, B2) for hypoechoic targets and masses (C1, C2) for hyperechoic targets for quantitative evaluation using the tissue phantom model.

Figure 10 shows the B-mode image simulation results which were performed to ascertain the degree of improvement of image quality in STMF technique using the orthogonal chirps which were designed for simultaneously transmitting in CMUT array. The main goal for this preliminary study is to demonstrate how the orthogonal chirp coded excitation for STMF technique would affect contrast and spatial resolution before the CMUT probe is implemented into the commercial ultrasound system. 

Table 2 summarizes the quantitative evaluation and shows how much the STMF technique using orthogonal chirp coded excitation improves the image quality in terms of contrast and spatial resolution. For the quantitative comparison of the spatial and contrast resolution, the typical evaluation metrics of the −6 dB main-lobe beam-width in lateral and axial direction, and CNR were used. CNR was computed by:

(8) $$CNR=\frac{\left|\mu_{i}-\mu_{o}\right|}{\sqrt{\sigma_{i}^{2}+\sigma_{o}^{2}}}$$

where $\mu_{i,o}$ and $\sigma_{i,o}^{2}$ are the mean intensities and variances of image pixels inside and outside of 4 targets (C1, C2, B2, B1) as shown in Figure 10h. The mean value of four different outside regions which correspond to four dotted regions outside the targets in Figure 10h was used to compute the mean intensity and variance. For the penetration, the theoretical analysis based on the gain in peak amplitude obtained in the experiment was used.

Figure 10a shows the B-mode image of conventional SF method with Tx focus of 40 mm using a short pulsed-wave, in which two cycles 4.66 MHz sine wave with Hanning window was used. The wavelength of the pulsed-wave was 0.66 mm and the −6 dB width (FWHM) of this pulse was 0.34 mm. The −6 dB axial beam-width of the point targets P1~P6 was measured as 0.42 mm~0.48 mm. As the short pulsed-wave was convolved with the impulse response of transducer, the emitted waveform through the transducer could be elongated. Moreover, the Green’s function ($e^{jkR}/R)$ and obliquity factor (z/R) are still effective in the beam-field pattern and the attenuation due to these factors affects the radiation pattern in ultrasound wave propagation where k is the wave number given by 2π/λ, λ is the wavelength, R is the distance between the source and target in the field, and z is the position in axial direction of the target [51,52]. Thus the axial beam width can be increased depending on the depth and compared to the original wavelength of pulsed-wave.

It was demonstrated that the major benefit adopting STMF technique using orthogonal chirp coded excitation in CMUT was the improvement of contrast resolution. As shown in Figure 9b–g and Table 2, the contrast resolution in STMF technique was improved at most regions. Especially the improvement at far field regions (B1) was much higher than that in other regions. The highest improvement was also 74.7% at the region B1 in Figure 10g. Even though C2 region (40 mm) was the focused depth of SF method, CNR values measured in all SMTF methods were higher than that of SF method. Figure 9b–g also showed that the STMF method enhances the contrast resolution in every region regardless of the combination of focused depth for orthogonal chirps.

At the C1 region (30 mm) which was the focused depth of Figure 9c,d,f, 11.2% improvement of CNR in Figure 9c,d was lower than 30.0% CNR improvement in Figure 10f. As the higher frequency chirp was focused at C1 region in Figure 9f, contrast resolution was improved more than 2.5 times compared to Figure 9c,d in which lower frequency chirp was used for focusing at C1 region. This was applied to the comparison between Figure 9b,e in which lower frequency chirp was focused at 20 mm, and Figure 10g in which higher frequency chirp was focused at 20 mm. CNR improvement of 38.1% in Figure 10g was higher than 16.8% CNR improvement in Figure 9b,e. However, CNR improvement of Figure 10g was decreased at C2 region (40 mm) and almost same as the CNR improvement values in Figure 9b,e. As higher frequency had lower penetration, the focusing effect at 40 mm was weakened. The focusing effect in Figure 10f lasted at C2 region (40 mm) as the focus depth was 30 mm. Thus the improvement of CNR in Figure 10f at C2 region was still higher than others, especially than that of Figure 10g. At B2 region (60mm), the CNR improvement in Figure 9c,e was the highest as chirp2 (*c*_2_) was focused at this depth in those Figures. Compared to Figure 10g in which chirp1 (*c*_1_) was focused at 60 mm, CNR improvement in Figure 9c,e in which higher frequency chirp was used for focusing was higher. Similarly, at B1 region (70 mm), Figure 10g in which lower frequency chirp was used for focusing at 60 mm had the highest CNR improvement.

In terms of contrast resolution based on the evaluation of CNR values, Figure 10g was the best. Furthermore, considering higher frequency provides lower penetration, higher frequency at near field and lower frequency at far field can be an optimized combination to utilize multiple frequency components in STMF using orthogonal band-divided signals. However, in a viewpoint of overall qualitative evaluation from the B-mode images combined with the quantitative evaluation, Figure 10e would be the best as the higher frequency chirp focused at 60 mm improves the image quality of deeper regions.

The lateral resolution of B-mode images adopting the STMF technique was improved at every depth (P1~P7) except P3 (40 mm) which was the focus depth of SF method. At P1 target (20 mm) which was the focused depth for low frequency chirp *c*_1_ used in Figure 9b,e, and high frequency chirp *c*_2_ used in Figure 10g, the improvement in lateral resolution of Figure 9b,e and Figure 10g were 45.0% and 67.5%, respectively. As higher frequency chirp was used in Figure 10g, the improvement was the highest. It was remarkable that 52.5% improvement in lateral resolution of Figure 10f was higher than that of Figure 9b,e. This was because higher frequency chirp improves the lateral resolution, in which relationship is given by $\Delta x\propto f_{num}\cdot c/f_{0}$ where Δx is the main-lobe width of lateral beam pattern, $f_{num}$ is the f-number, c is the sound speed, and $f_{0}$ is the center frequency of the ultrasound wave. At P2 target (30 mm), this phenomenon was observed as well. Even though the focus depth of Figure 9c,d,f was all 30 mm, 77.1% improvement of lateral resolution of Figure 10f was higher than 40.0% of Figure 9c,d. At P3 target (40 mm), as this was the focus depth of SF method, no improvements were shown in the lateral resolution in all STMF methods. From P4 (50 mm) to P6 (70 mm), the similar pattern in improvement of lateral resolution could be observed. For the P4 target (50 mm), Figure 9b,d showed the highest improvement in lateral resolution as the high frequency chirp *c*_2_ was used for focusing at 50 mm (P4), which was better than Figure 10f in which lower frequency chirp *c*_1_ was focused at 50 mm. At P5 target (60 mm), Figure 9c,e provided the most improved lateral resolution due to the same reason as before. At P6 target (70 mm), Figure 9c,e where the focus depth was the closest showed the highest improvement in lateral resolution, similarly.

To maintain the axial resolution in this simulation, orthogonal chirps were designed to have almost same bandwidth as that of the pulsed-wave. The −6 dB frequency bandwidth of the two orthogonal chirps were 2.46 MHz ($c_{1}\left(t\right):$ 1.95 MHz~4.41 MHz) and 2.47 MHz ($c_{2}\left(t\right):$ 7.36 MHz~4.89 MHz), respectively while the −6 dB frequency bandwidth of pulsed wave was 2.52 MHz as shown in Table 1. However, it was observed that the frequency bandwidth of the chirps after matched filtering was narrowed by 22.6% for the low frequency chirp *c*_1_ and 18.8% for the high frequency chirp *c*_2_ as described in Section 4.3.1 that experimental results for two sub-band chirps were shown. The degradation of frequency bandwidth could elongate the length of compressed wave as discussed in Section 4.3.2 and could result in the decline of the axial resolution. Nevertheless, the degradation of axial resolution shown in this study was smaller than previously introduced results to investigate STMF technique using orthogonal quadratic chirp signals [26]. Compared to chirp coded excitation using new dynamic complex baseband pulse compression recently introduced [53], the axial resolution of STMF method using frequency band-divided chirps was better. As the frequency bandwidth of CMUT used in this work was broader than others, the frequency bandwidth of orthogonal chirps could be designed as widely as possible to be comparable to the frequency bandwidth of pulsed-wave.

A phantom and in vivo experiment need to be performed to investigate the effects of inhomogeneity and nonlinearity in tissues and the effectiveness in clinical application though the simulation results obtained using the proven and well-established simulator are adequate to ascertain the features and expected benefits of orthogonal chirp coded excitation in the CMUT array.

## 5. Conclusions

The main goal of this work was to validate that the 2-D CMUT-on-ASIC array can maximize the advantage of FDMA based STMF technique using orthogonal chirps in both simulation and experimental studies. In this preliminary study, experiments were conducted to show that simultaneously transmitted orthogonal chirps designed within the frequency bandwidth of CMUT were successfully separated and compressed into two short pulses from the received signal by performing the pulse compression and separation with the correlators. The experimental results validate that the orthogonal chirp coded excitation in the 2-D CMUT-on-ASIC array is feasible without any distortion in frequency characteristics of transmitted chirps. It has been observed that the frequency bandwidth after matched filtering narrows in the experiment and this should be considered in the design of orthogonal chirps used for the STMF technique. The simulation study was also conducted to quantitatively and qualitatively evaluate the image quality of STMF technique using orthogonal chirp coded excitation in CMUT array. It has been shown that orthogonal chirp coded excitation for the STMF technique in CMUT array provides more improved quality of images in terms of contrast resolution and lateral resolution than previous works. It has been demonstrated that higher frequency chirp provides higher contrast and spatial resolution, and the various combination of Tx foci for orthogonal chirps is feasible for STMF technique in CMUT array. The simulation results support that the image quality can be improved in the STMF technique using two orthogonal chirps designed to be utilized in CMUT array. It has been shown that the axial resolution was better than any other results which were previously introduced as the axial beam-width is not sacrificed due to the wide bandwidth characteristic of the CMUT array in both simulation and experimental results. Accordingly, the wide bandwidth characteristic of CMUT array can maximize the benefit of STMF technique using frequency band-divided sub-band signals without sacrificing the frame rate and axial resolution. The phantom and in vivo study should be performed after the ultrasound system and the CMUT-ASIC array is integrated and the arbitrary waveform generation for orthogonal chirp coded excitation in the integrated system is enabled.

## Figures and Tables

**Table 1:** Simulation parameters.

Parameters	Value
Number of Tx/Rx channels	64
Sampling frequency	80 MHz
Element pitch	0.25 mm
Transducer array type	Linear array
Number of elements in transducer array	192
Ultrasonic wave propagation speed	1540 m/sec
Attenuation	0.3 dB/MHz/cm
Center frequency of pulsed wave	4.66 MHz
−6 dB bandwidth of pulsed wave	2.52 MHz
Transmission focus depth of pulsed wave	40 mm
Center frequency of chirp $c_{1}\left(t\right)$	3.18 MHz
−6 dB bandwidth of chirp $c_{1}\left(t\right)$	2.46 MHz
Center frequency of chirp $c_{2}\left(t\right)$	6.13 MHz
−6 dB bandwidth of chirp $c_{2}\left(t\right)$	2.47 MHz
Tx foci of two chirps: $c_{1}\left(t\right)$/$c_{2}\left(t\right)$ [mm]	20/50, 30/60, 30/50, 20/60, 50/30, 60/20

**Table 2:** Quantitative evaluation and improvement in % of contrast resolution (CNR) and spatial resolution (−6 dB beam width in lateral and axial direction).

Method			SF	STMF					
			(a)	(b)	(c)	(d)	(e)	(f)	(g)
Focus [mm]			40	$c_{1}\left(t\right)$: 20	30	30	20	50	60
				$c_{2}\left(t\right)$: 50	60	50	60	30	20
Contrast resolution: CNR (upper row) and improvement in % (lower row)	C1 (30 mm)		3.57	4.17	3.97	3.97	4.17	4.64	4.93
			-	16.8	11.2	11.2	16.8	30.0	38.1
	C2 (40 mm)		3.89	4.76	5.01	4.76	5.01	5.12	4.80
			-	22.4	28.8	22.4	28.8	31.6	23.4
	B2 (60 mm)		3.59	3.95	4.43	3.95	4.43	3.74	4.12
			-	10.0	23.4	10.0	23.4	4.2	14.8
	B1 (70 mm)		2.41	3.02	3.79	3.02	3.79	2.95	4.21
			-	25.3	57.3	25.3	57.3	22.4	74.7
Lateral and axial resolution [mm]: −6 dB main-lobe beam-width (upper row) and improvement in % (lower row)	P1 (20 mm)	Lateral	0.80	0.44	0.78	0.78	0.44	0.38	0.26
			-	45.0	2.5	2.5	45.0	52.5	67.5
		Axial	0.42	0.49	0.48	0.48	0.49	0.48	0.45
			-	−16.7	−14.3	−14.3	−16.7	−14.3	−7.1
	P2 (30 mm)	Lateral	1.18	0.78	0.72	0.72	0.78	0.27	0.74
			-	33.9	40.0	40.0	33.9	77.1	37.3
		Axial	0.43	0.49	0.49	0.49	0.49	0.46	0.48
			-	−14.0	−14.0	−14.0	−14.0	−7.0	−11.6
	P3 (40 mm)	Lateral	0.72	0.88	1.22	0.88	1.22	0.76	1.92
			-	−22.2	−69.4	−22.2	−69.4	−5.6	−166.7
		Axial	0.43	0.51	0.51	0.49	0.51	0.48	0.52
			-	−18.6	−18.6	−14.0	−18.6	−11.6	−21.0
	P4 (50 mm)	Lateral	1.44	0.64	0.98	0.64	0.98	0.85	1.38
			-	55.6	31.9	55.6	31.9	41.0	4.2
		Axial	0.43	0.49	0.48	0.49	0.48	0.49	0.52
			-	−14.0	−11.6	−14.0	−11.6	−14.0	−21.0
	P5 (60 mm)	Lateral	2.28	1.24	0.86	1.24	0.86	1.36	1.28
			-	45.6	62.3	45.6	62.3	40.4	43.9
		Axial	0.47	0.53	0.50	0.53	0.50	0.54	0.53
			-	−12.8	−6.4	−12.8	−6.4	−14.9	−12.8
	P6 (70 mm)	Lateral	2.76	1.91	1.04	1.91	1.04	1.92	1.67
			-	30.8	62.3	30.8	62.3	30.4	39.5
		Axial	0.48	0.46	0.47	0.46	0.47	0.50	0.49
			-	4.2	2.1	4.2	2.1	−4.2	−2.1
